# Metagenomics insights into bacterial diversity and antibiotic resistome of the sewage in the city of Belém, Pará, Brazil

**DOI:** 10.3389/fmicb.2024.1466353

**Published:** 2024-11-19

**Authors:** Sérgio Ramos, Edivaldo Júnior, Oscar Alegria, Elianne Vieira, Sandro Patroca, Ana Cecília, Fabiano Moreira, Adriana Nunes

**Affiliations:** ^1^Laboratory of Genomics and Bioinformatics, Center of Genomics and Systems Biology, Federal University of Pará, Belém, Brazil; ^2^Oncology Research Center, João de Barros Barreto Hospital, Federal University of Pará, Belém, Brazil; ^3^Laboratory of Leishmaniasis, Parasitology Section, Evandro Chagas Institute, Ananindeua, Brazil; ^4^Arbovirology and Hemorrhagic Fevers Section, Evandro Chagas Institute, Ananindeua, Brazil

**Keywords:** metagenome, sewerage, microbial diversity, resistome, antibiotic resistant bacteria

## Abstract

**Introduction:**

The advancement of antimicrobial resistance is a significant public health issue today. With the spread of resistant bacterial strains in water resources, especially in urban sewage, metagenomic studies enable the investigation of the microbial composition and resistance genes present in these locations. This study characterized the bacterial community and antibiotic resistance genes in a sewage system that receives effluents from various sources through metagenomics.

**Methods:**

One liter of surface water was collected at four points of a sewage channel, and after filtration, the total DNA was extracted and then sequenced on an NGS platform (Illumina® NextSeq). The sequenced data were trimmed, and the microbiome was predicted using the Kraken software, while the resistome was analyzed on the CARD webserver. All ecological and statistical analyses were performed using the. RStudio tool.

**Results and discussion:**

The complete metagenome results showed a community with high diversity at the beginning and more restricted diversity at the end of the sampling, with a predominance of the phyla Bacteroidetes, Actinobacteria, Firmicutes, and Proteobacteria. Most species were considered pathogenic, with an emphasis on those belonging to the *Enterobacteriaceae* family. It was possible to identify bacterial groups of different threat levels to human health according to a report by the U.S. Centers for Disease Control and Prevention. The resistome analysis predominantly revealed genes that confer resistance to multiple drugs, followed by aminoglycosides and macrolides, with efflux pumps and drug inactivation being the most prevalent resistance mechanisms. This work was pioneering in characterizing resistance in a sanitary environment in the Amazon region and reinforces that sanitation measures for urban sewage are necessary to prevent the advancement of antibiotic resistance and the contamination of water resources, as evidenced by the process of eutrophication.

## 1 Introduction

For many years, antibiotic therapy has been established as an effective form of treatment for various bacterial infections, especially during the mid-20th century with the discovery of penicillin (Fleming, 1929). However, inadequate medical prescription and the indiscriminate use of antibiotics have become recurrent practices in the global population, resulting in high selective pressure on various bacterial strains and initiating the antimicrobial resistance (AMR) crisis (Low, 2002).

Currently, AMR consists of a wide variety of antibiotic resistance genes (ARGs), which are found in bacterial strains from diverse environments such as soil (Djenadi et al., 2018), hospitals (Mulvey and Simor, 2009), pharmacies (Obayiuwana et al., 2018), food (Founou et al., 2016), and aquatic systems (Marti et al., 2013). Both resistance genes and antibiotics are introduced into the environment through the release of sanitary effluents from urban areas and rural agricultural activities, altering the composition and ecological dynamics of the local microbiome and contributing to the diversity of ARGs (Wright, 2010). Consequently, sewage networks form an important reservoir for horizontal gene transfer (HGT) in resistant bacterial ecosystems due to the presence of mobile genetic elements (MGEs) such as plasmids, transposons, and integrons (Wu et al., 2019; Pärnänen et al., 2019).

The implementation of wastewater treatment plants (WWTP) that promote the sanitization of urban and rural liquid effluents is an alternative for the elimination of antibiotic-resistant bacteria and, consequently, the containment of AMR (V. M. Starling et al., 2021). The high availability of decomposing organic matter combined with the presence of antibiotic residues and other molecules in sewage waters makes this environment conducive to the transfer of ARGs among bacteria (Atashgahi et al., 2015). Among some human pathogenic taxa commonly cited in metagenomic studies of wastewater are *Escherichia coli*, *Klebsiella pneumoniae*, *Pseudomonas aeruginosa*, *Staphylococcus aureus*, *Shigella* spp., *Salmonella* spp., *Vibrio* spp., *Acinetobacter* spp., and *Enterococcus* spp. (Pärnänen et al., 2019; Fouz et al., 2020).

Therefore, to understand and evaluate the microbiome present in sewage systems, studies have used the metagenomic approach to elucidate knowledge gaps in environmental microbiology, as it is a cultivation-independent technique (Rowe et al., 2017; Hendriksen et al., 2019; Schloss and Handelsman, 2003). This approach allows for the exploration of the microbial framework present in the Brazilian Amazon region which, despite its vast extent of water resources, lacks studies related to AMR (Freitas et al., 2019).

Thus, the present study aimed to characterize the bacterial diversity and gene repertoire associated with antibiotic resistance in a sanitary sub-basin in the municipality of Belém, which lacks a connected WWTP and discharges its effluents through a collector channel directly into a natural resource, the Guamá River. As mentioned earlier, sanitary effluents alter the aquatic microbial ecosystem and favor the emergence of AMR and ARGs. To assess the presence of these in this sub-basin, metagenomics was applied to also monitor how microbial communities and the antibiotic resistome are characterized in this type of water resource in the city.

## 2 Materials and methods

### 2.1 Water sampling

Water samples were collected once in February 2021 from an open-air sewage collector channel, with an extension of 934 meters, belonging to the Una sub-basin, Belém, Pará, Brazil. Four collection points were marked, which were spaced at equal distances along the length of the collector channel: P1 (1.448167 S 48.486778 W); P2 (1.445972 S 48.488389 W); P3 (1.443833 S 48.489972 W); P4 (1.442028 S 48.492194 W). These collection sites were strategically selected, as they are located close to sanitary waste disposal pipes.

For the metagenomic analysis, biological triplicate sampling was performed, collecting 1 L of surface liquid effluent (1 m) at each point using appropriate and pre-sterilized materials, in which the replicates were named P1R1, P1R2, P1R3 (for point P1) and so on. For the physicochemical analyses, 2 L of wastewater were obtained from a pool of 500 mL collected at each point. Due to budgetary constraints, we were unable to request analysis for each collection site. The samples were stored and labeled in sterile 1 L polypropylene bottles and transported in a climate-controlled cooler to the laboratory who provided outsourced services for physicochemical analysis.

The evaluation of physicochemical and microbiological parameters was carried out by Multianálises S/S, following an individual protocol and assessing the parameters: dissolved oxygen, salinity, pH, conductivity, chemical oxygen demand, biochemical oxygen demand, total coliforms, and *Escherichia coli*. Due to the nature of the sample being wastewater, the results of these parameters could not be compared with those stipulated in Resolution No. 357/2005 of the National Environmental Council (CONAMA), as there is no legislation addressing the quality of this type of water resource (Brazil, 2005).

### 2.2 Metagenomic approach

#### 2.2.1 Sample processing, total microbial DNA (TM-DNA) extraction and quality

The sampling at each point, considering the collected triplicates, resulted in a total of 12 samples, with the contents (1 L) filtered using a 0.22 μm pore nitrocellulose membrane (MF-Millipore®) connected to a vacuum pump. The membrane was replaced with a new one when saturated, until the entire content of each sample was exhausted. The membranes were placed in Falcon tubes (50 mL) containing saline-Tris-EDTA solution for washing, aided by a shaker (120 RPM/12H) at room temperature. After washing, the tube contents were centrifuged (9,500 RPM/10′) to form a pellet and stored at −20°C.

DNA extraction was performed using the DNeasy PowerLyzer PowerSoil kit (QIAGEN), following the manufacturer’s instructions. The quality of the obtained TM-DNA was verified using a NanoDrop Lite (Thermo Fisher Scientific), accepting samples with a concentration and purity level of 50 ng/μL and 1.8 to 2.0 nm, respectively. The integrity of the TM-DNA was assessed by 1% agarose gel electrophoresis with the addition of 0.1 μL of ethidium bromide, visualized under ultraviolet light.

### 2.3 Next generation sequencing and metagenome community analysis

The whole-metagenome shotgun approach was chosen to characterize the microbiome, in which the library preparation kit for metagenomic sequencing of the samples was the Nextera XT DNA Library Preparation Kit (Illumina), and the technique utilized was the NextSeq 550 System (Illumina) with a paired-end approach (2×150 bp). Following this, the quality of the total reads was analyzed using the FastQC tool, and a Phred quality score cutoff of 33 was adopted. Sequences with lower quality were removed and filtered using the Trimmomatic program, admitting sequences with a minimum length of 200 bp.

**Figure 1:**
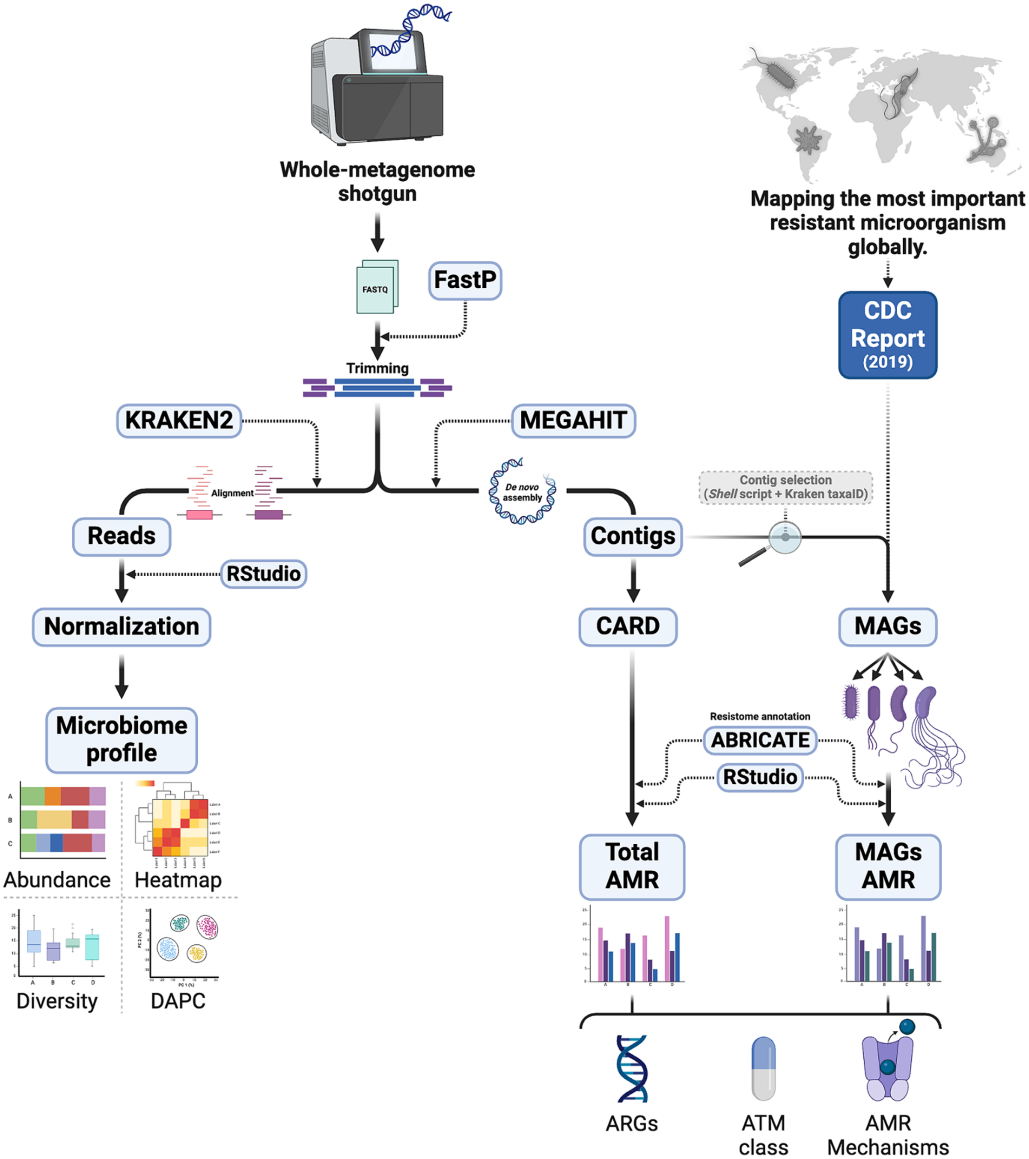
Methodological steps of sample processing for microbial profiling (left) and resistome (right), including analysis of MAGs of pathogens reported by CDC. DAPC, discriminant analysis of principal components; CARD, the Comprehensive Antibiotic Resistance Database; CDC, Centers for Disease Control and Prevention; MAG, metagenome-assembled genome; AMR, antimicrobial resistance; ARG, antimicrobial resistance gene; ATM, antimicrobial.

The identification of operational taxonomic units (OTU) from the reads was performed using Kraken 2 software (Wood et al., 2019), which generated report files containing the number of reads and the taxonomic description of each taxon (see Figure 1). The Pavian package in RStudio (RStudio Team, 2020) assisted in producing tables for ecological analyses. The standardization of the number of reads was achieved by the “prop.table” function, applying a conditional proportion based on the sum of columns for each sample, and multiplying by one million. The clustering of samples from each collection site was evaluated using discriminant analysis of principal components (DAPC) (Jombart, 2008; Jombart et al., 2010) through the “adegenet” package, and permutational multivariate analysis of variance (PERMANOVA) (Anderson, 2017) was performed using the “vegan” package. Richness (S) and diversity analyses, utilizing the Shannon (H′) indices, were also conducted using the “vegan” library, with rarefaction curves based on Hill numbers generated by the iNEXT package (Hill, 1973). All statistical tests were executed within the same program.

For abundance analyses, the most representative taxa among phyla, families, and genera were selected, and the corresponding graphs were produced using the “ggplot2” library in RStudio. Heatmap construction was performed using the “Pheatmap” package in the same program, employing “correlation” and “euclidean” as clustering distances and the “complete” method, which statistically hierarchizes the most similar treatments, considering the sampling points as independent variables (Figure 1). To illustrate the distribution of pathogens in the study environment, eight pathogenic families of medical importance were selected based on various articles describing their presence in wastewater (Singh et al., 2020; Pärnänen et al., 2019; Fouz et al., 2020; Marutescu et al., 2023).

### 2.4 Metagenome assembly and resistome analysis

The assembly of metagenomic data was performed using the MEGAHIT software (Li et al., 2015), where the generated contigs were analyzed with the ABRICATE program utilizing the Comprehensive Antibiotic Resistance Database (CARD) (McArthur et al., 2013; Jia et al., 2017). The Resistance Gene Identifier (RGI) tool was employed using strict and perfect algorithms to identify antibiotic resistance genes (ARG) based on homology against 4,970 reference sequences. The parameters used followed the web server’s recommendations for metagenomic data, indicating “low” for sequence quality and considering ARG with a minimum of 80% identity and 95% coverage, respectively. At the end, a report was generated containing the gene name/ID, gene family, coverage, identity, resistance mechanism, and antibiotic class to which the gene is linked. The Centers for Disease Control and Prevention (CDC) Threat Report was used as a guide to select the four genera of bacterial pathogens with serious health threat levels (Centers for Disease Control and Prevention, 2019). All contigs related to the selected genera were retrieved through a shell script and using the taxaID of the respective OTUs from the Kraken2 reports of each sample. Subsequently, the resistome of these metagenome-assembled genomes (MAGs) was predicted by the same method above for each pathogen. More details of this step are shown in Figure 1.

## 3 Results

### 3.1 Physical, chemical and microbiological analysis

Regarding the physicochemical findings obtained from the pooled sample taken from the sewage channel, the parameters considered were salinity, pH, electrical conductivity, dissolved oxygen (DO), chemical oxygen demand (COD), and biochemical oxygen demand (BOD). Among these, the pH was found to be highly acidic (1.17 at 25°C), and the conductivity was measured at 407 μS/cm at 25°C. For the microbiological parameters, total coliforms and *Escherichia coli* were quantified and found to be present in the sample (Supplementary Table 1).

### 3.2 Sequencing and metagenomic community analysis

The metagenome of microbial communities present in the wastewater yielded, on average, approximately 23 million (± 4,417,343.649), 21 million (± 8,023,626.86), 24 million (± 14,329,491.92), and 22 million (± 14,515,690.23) reads at sampling points P1, P2, P3, and P4, respectively (see Supplementary Table 2 for details).

**Figure 2:**
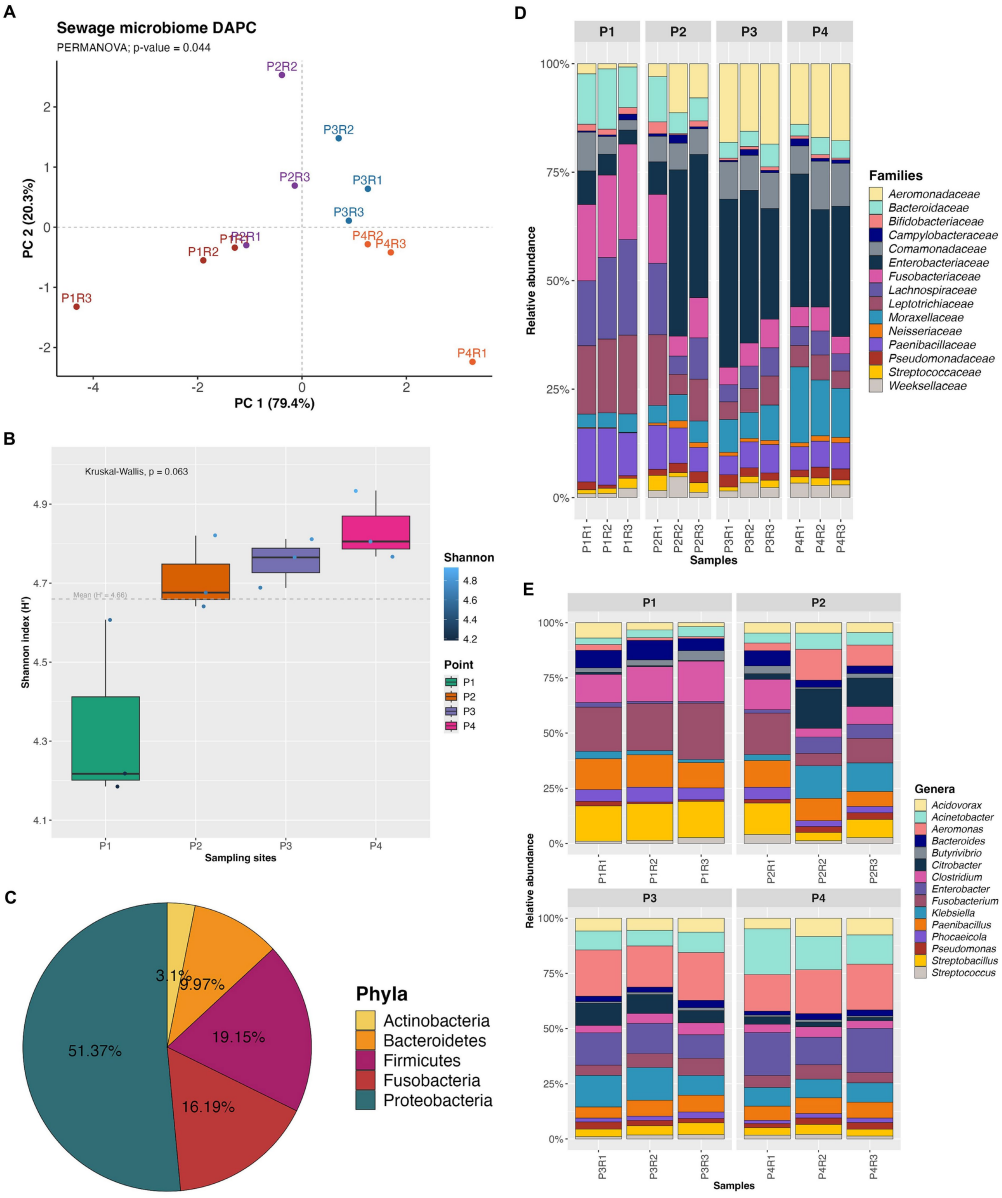
Discriminant analysis of principal components (DAPC) **(A)**, Shannon alpha diversity **(B)**, relative abundance of the phyla **(C)**, families **(D)** and genera **(E)** most representative of the microbiome.

The DAPC performed on the number of reads of OTUs in each sample revealed that the clusters of the sites were close to each other, indicating a certain similarity in abundances between the sampling points. However, the DAPC groups showed significant differences (PERMANOVA; *p*-value = 0.044) between the sampling sites (Figure 2A). Alpha diversity generated from 7,556 OTUs at species level showed that Shannon index values increased from the first to the last collection point, but there was no statistically significant difference between the sampling sites (Kruskal-Wallis; *p*-value = 0.063) (Figure 2B). Other ecological parameters related to the metagenomic communities can be found in Supplementary Table 3.

The Bacteria domain was the most predominant across all samples, with the most representative phyla being Proteobacteria (P1: 4.79%; P2: 12.11%; P3: 17.03%; P4: 17.42%), Firmicutes (P1: 7.90%; P2: 5.05%; P3: 3.15%; P4: 3.03%), Fusobacteria (P1: 7.47%; P2: 4.24%; P3: 2.37%; P4: 2.09%), Bacteroidetes (P1: 3.71%; P2: 2.57%; P3: 1.83%; P4: 1.83%), and Actinobacteria (P1: 1.03%; P2: 0.94%; P3: 0.55%; P4: 0.56%) (Figure 2C).

The five most predominant families were *Enterobacteriaceae* (20.25%), *Aeromonadaceae* (9.32%), *Fusobacteriaceae* (8.29%), *Lachnospiraceae* (8.11%), and *Leptotrichiaceae* (7.90%) (Figure 2D). Regarding the ten most abundant genera overall, the highlights are *Aeromonas* (9.23%), *Fusobacterium* (8.28%), *Enterobacter* (6.78%), *Paenibacillus* (6.62%), *Acinetobacter* (6.26%), *Klebsiella* (6.15%), *Streptobacillus* (5.97%), *Clostridium* (5.85%), *Citrobacter* (4.02%), and *Acidovorax* (3.89%) (Figure 2E).

**Figure 3:**
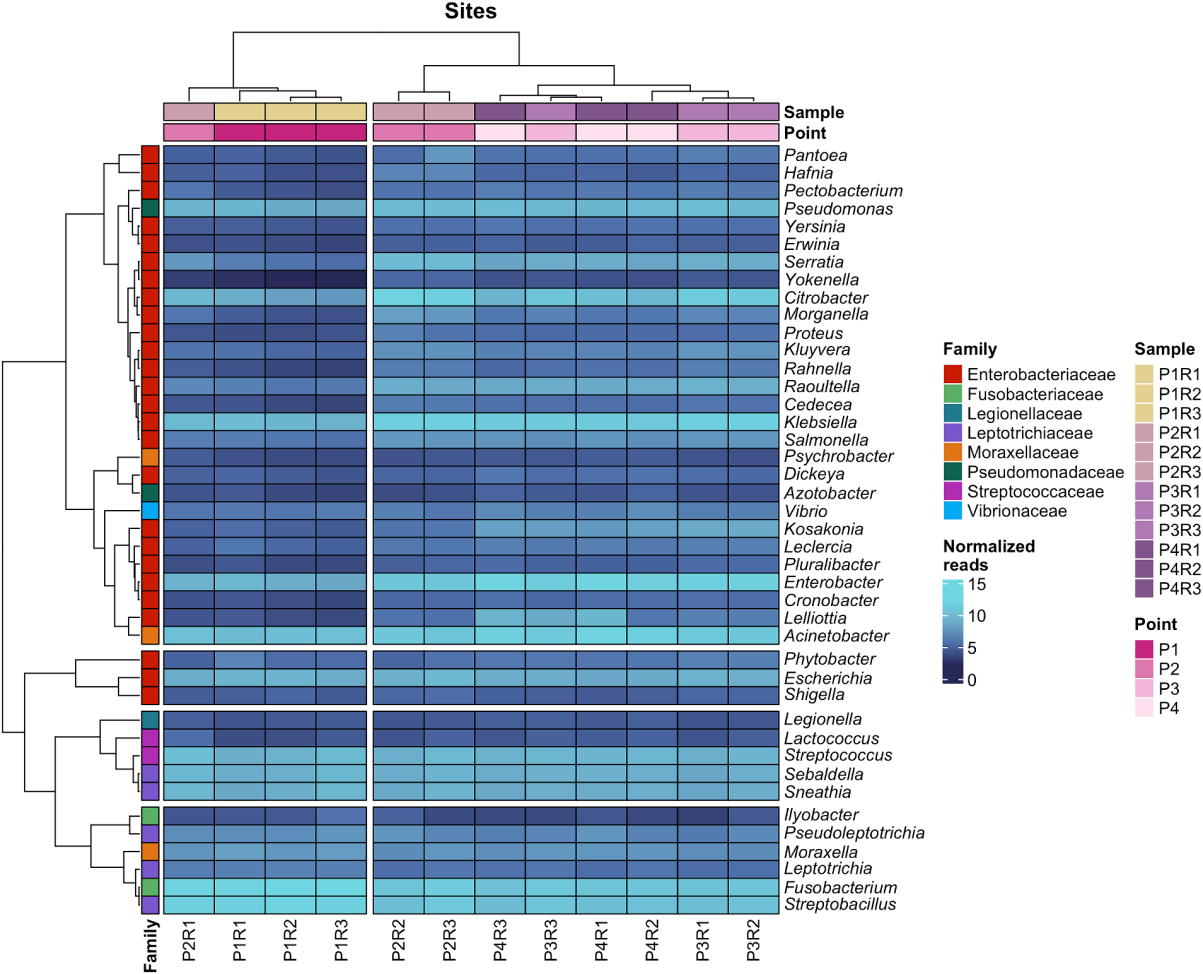
Heatmap with the abundance (in log scale) of bacterial genera of medical importance and the respective families that compose them.

Finally, pathogenic bacteria at the family taxonomic level had their respective genera highlighted in a heatmap showing the distribution of their abundances, on a logarithmic scale, in the water body. Additionally, taxa with a similar number of reads between samples and between genera are clustered (Figure 3).

*Enterobacteriaceae* included 26 genera, which are present in two groups, one containing *Escherichia* and *Shigella*, and another larger group encompassing various pathogenic genera such as *Acinetobacter*, *Klebsiella*, *Enterobacter*, *Fusobacterium*, and *Streptobacillus*. It is noted that samples with the lowest number of reads, P1R2, P1R3, and P2R1, clustered separately from the others, with this distinction being evident for most *Enterobacteriaceae* genera. The replicate P1R1 did not present a satisfactory number of reads for these microbial groups and therefore is not included in this analysis.

When assessing whether the microbiome of the sewage channel could be significantly influenced by the microbial load of hospital sanitary effluents, the abundances of pathogenic bacteria at point P4 (located between two hospitals) did not show significant differences compared to other collection points (Kruskal-Wallis; *p*-value = 0.96).

### 3.3 Resistome analysis

The contigs generated from the metagenomic data assembly achieved N50 values above 600, with the complete description of the assembly parameters available in Supplementary Table 2. From the contigs, CARD identified 92, 120, 118, and 102 ARGs at points P1, P2, P3, and P4, respectively. Additionally, around 33% of the genes at P1 conferred resistance to multiple drugs, while the points P2 and P3, approximately 32 and 35%, respectively, while P4 had around 22% MDR genes.

**Figure 4:**
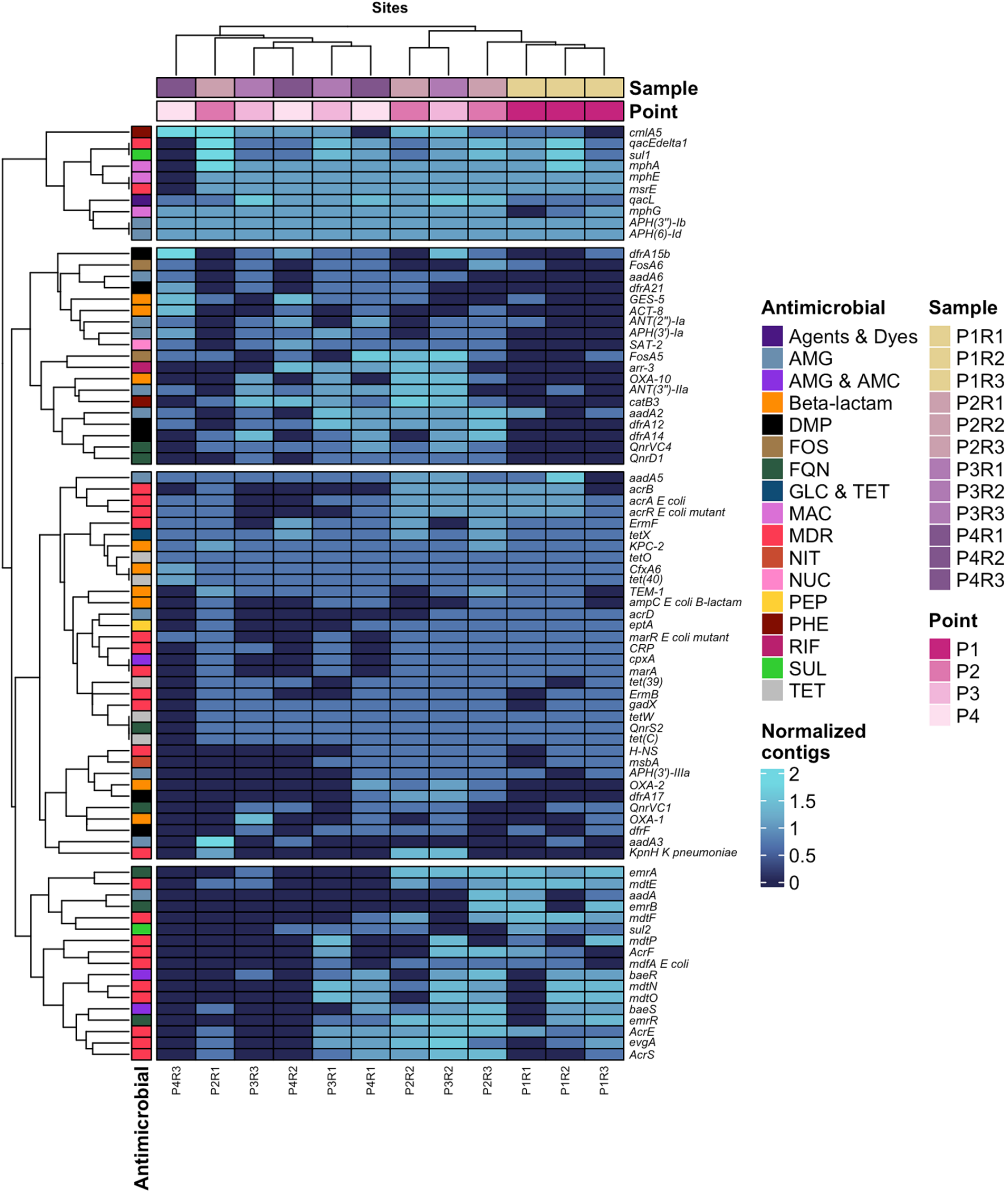
Heatmap of the 80 most predominant ARGs in the resistome and their respective antimicrobials to which they confer resistance. AMG, Aminoglycoside; AMC, Aminocoumarin; DMP, Diaminopyrimidine; Agents and Dyes, Disinfecting agents and intercalating dyes; FQN, Fluoroquinolone; FOS, Fosfomycin; GLC, Glycylcycline; TET, Tetracycline; MAC, Macrolide; MDR, Multidrug resistant; NIT, Nitroimidazole; NUC, Nucleoside; PEP, Peptide; PHE, Phenicol; RIF, Rifamycin; SUL, Sulfonamide; TET, Tetracycline.

From the 80 most abundant ARGs, a heatmap was created, identifying the presence of four gene clusters distributed across the points, namely: Group 1: *cmlA5* – *aph(6)-ld*; Group 2: *dfrA15b* – *qnrD1*; Group 3: *aadA5* – *kpnH K pneumoniae*; Group 4: *emrA* – *AcrS* (Figure 4). Additionally, four gene clusters were identified, with the first three showing the most extensive distribution of genes along the sewage channel. However, the report generated by CARD also revealed numerous genes that individually confer resistance to multiple drug classes, all of which were classified under the MDR category, such as: *ermF, qacEdelta1*, *msrE*, *mdtE*, *acrB*, among others of great medical importance conferring resistance to macrolide, lincosamide, acridine, fluoroquinolone, penam and streptogramin.

The second and third groups of genes expressed heterogeneity in abundance and are genetic variants of different families of *β*-lactamases that confer resistance to a variety of β-lactams, such as *bla_GES-5_*, *bla_KPC-2_*, *bla_OXA-1_*, *bla_OXA-10_*, *bla_OXA-2_*, *bla_TEM-1_*, and *bla_CFXA6_*. It is important to note that a total of 143 genes encoding β-lactamases were identified in the sewage channel resistome, although they are not shown in the heatmap (Figure 4).

**Figure 5:**
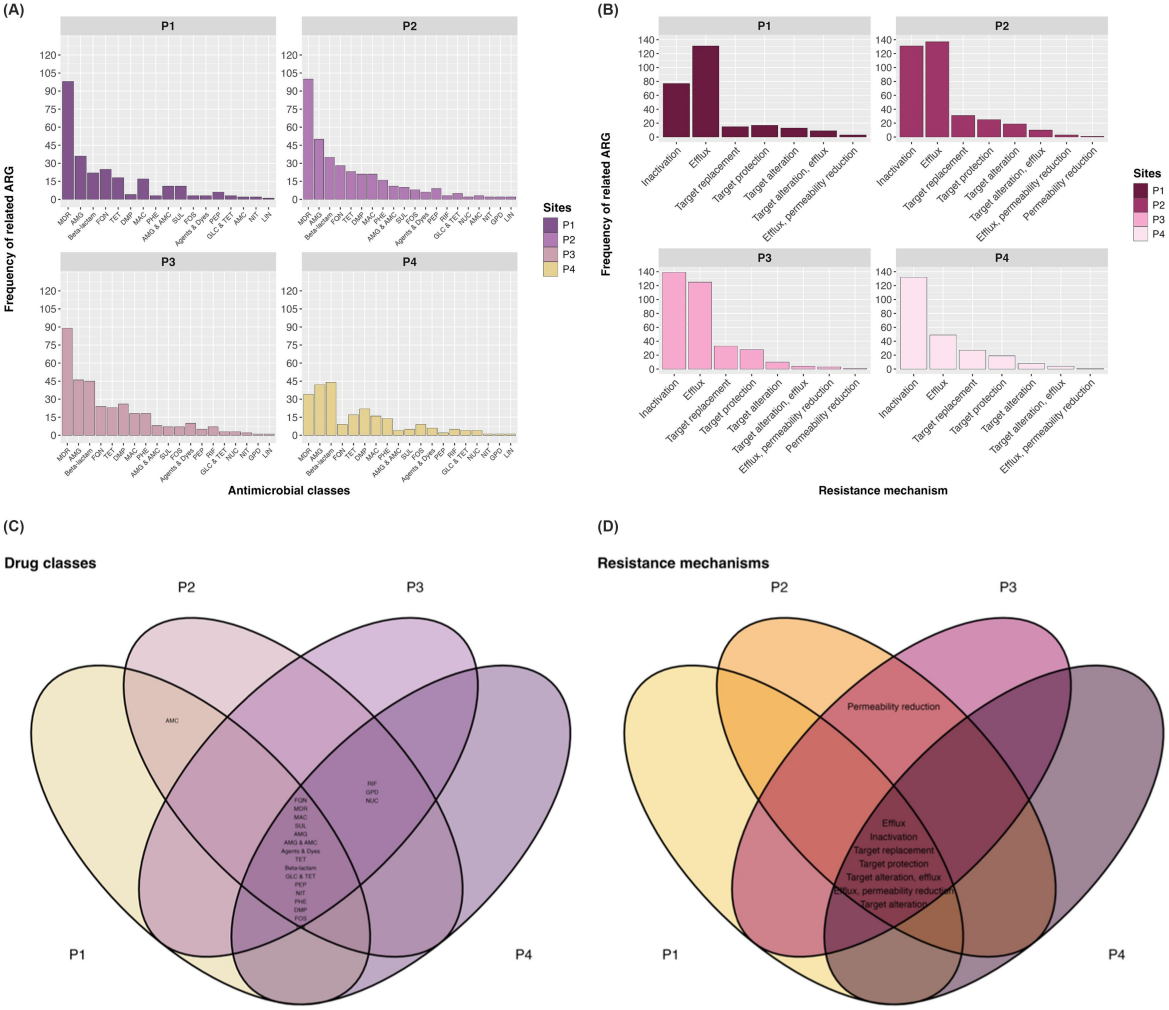
Frequency of ARGs (number of contigs) by antimicrobial class **(A)** and by type of resistance mechanism **(B)**. Venn plot for drug classes **(C)** and mechanisms **(D)** at each collection site. AMC, Aminocoumarin; AMG, Aminoglycoside; DMP, Diaminopyrimidine; Agents and Dyes, Disinfecting agents and intercalating dyes; FQN, Fluoroquinolone; RIF, Rifamycin; PEP, Peptide; FOS, Fosfomycin; GPD, Glycopeptide; TET, Tetracycline; GLC, Glycylcycline; LIN, Lincosamide; NUC, Nucleoside; MON, Monobactam; NIT, Nitroimidazole; MAC, Macrolide; PHE, Phenicol; SUL, Sulfonamide.

Another set of genes that stood out consists of ARGs associated with aminoglycosides (AMG), comprising 12 genes, being the second class with the largest number of genes in the top 80 most abundant ARGs depicted in Figure 5. Among these are *aph(6)-Id*, *aph(3″)-Ib*, *aadA2*, *aadA5* and *ANT(3″)-IIa*, being the most predominant of the AMG resistance genes.

The distribution of ARG abundance across antimicrobial classes in the sampled sites of the channel is depicted in Figure 5A, where categories such as MDR, aminoglycosides (AMG), fluoroquinolones (FNQ), and tetracyclines (TET) were generally the most predominant, except at point P4 where diaminopyrimidines (DMP) ranked third in ARG abundance, followed by TET. Regarding resistance mechanisms, from point P2 to P4, inactivation, efflux pump, and target alteration were the most important defense systems in terms of ARG numbers, while target protection was the third most abundant mechanism at P1 in this analysis (Figure 5B).

In the context of antimicrobial classes identified in the resistome, the Venn diagram illustrated in Figure 5C shows intersections where points have genes conferring resistance to, respectively, classes. The classes with the highest prevalence of ARGs were betas-lactams (37 different ARGs), followed by MDR (32), AMG (28), TET, and DMP, with 12 ARGs, respectively.

Regarding antimicrobial resistance mechanisms, the CARD web server detected a total of eight antibiotic evasion processes in the metagenomic data, as depicted in Figure 5D. It was found that 87.5% of resistance mechanisms were distributed across the four collection points, with inactivation being the predominant antibiotic evasion mechanism with the highest number of related ARGs (79), followed by efflux pump (44), and target alteration (14).

Investigating CDC reports on the landscape of AMR in the country, the governmental agency identified several significant microbial threats in the context of antibiotic resistance, including *Klebisiella pneumoniae*, *Acinetobacter baumannii*, *Pseudomonas aeruginosa*, and *Enterobacter cloacae*. These pathogens have been major contributors to increased hospitalizations and mortality due to rising antimicrobial resistance.

**Figure 6:**
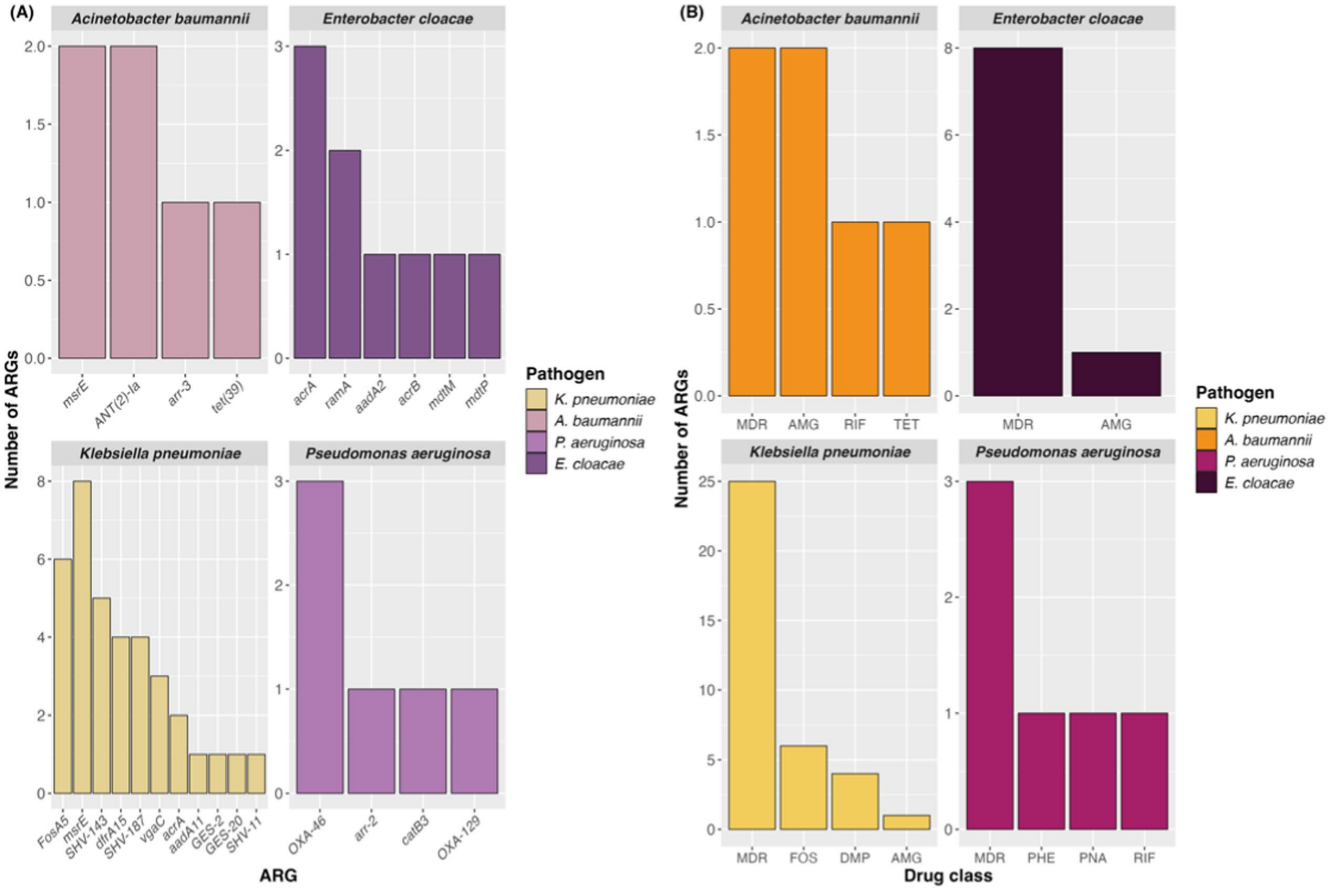
Number of ARGs **(A)** in each pathogen selected from the CDC report and antimicrobial classes related to them **(B)**. AMG, Aminoglycoside; DMP, Diaminopyrimidine; RIF, Rifamycin; PNA, Penam; FOS, Fosfomycin; TET, Tetracycline; PHE, Phenicol.

In this regard, the resistome generated for these agents exhibited a diverse profile of genes (Figure 6A), many of which encoded beta-lactamases and the majority of ARGs conferring multidrug resistance (Figure 6B). All evaluated pathogens showed an MDR profile, with *K. pneumoniae* notably demonstrating a broad repertoire of ARGs and the highest number of contigs associated with MDR ARGs.

## 4 Discussion

In the present study, we characterized the microbiome and resistome of an open sewage channel that receives liquid effluents from various sectors of the city of Belém, including domestic, hospital, industrial, and food service effluents. The accumulation of waste from diverse sources in the sanitary aquatic environment directly influences the selective pressure on the microbiome present there, contributing to the formation of a highly varied resistome, especially when this location does not receive adequate sanitary treatment (Mutuku et al., 2022).

### 4.1 Microbial communities analysis

It is important to note that the sewage channel under study lacks a wastewater treatment plant (WWTP). Geographically situated near the mouth of a river (known as Baía do Guajará), this sub-basin may receive contaminated effluents from the channel, thereby causing significant environmental impact on aquatic biota. As observed in the physical–chemical and microbiological parameters of the sewage effluents, the water quality of Baía do Guajará is continually compromised. This could potentially affect the health of riverside communities adjacent to the metropolis, who rely on river water for daily activities and consumption.

The presence of fecal coliforms indicates human feces in the water, carrying a high microbial load from the human gastrointestinal tract. This can disseminate pathogenic microorganisms in the aquatic environment, increasing oral-fecal transmission of diseases, especially in the absence of WWTPs that effectively treat highly polluted effluents such as those from hospitals (McLellan and Eren, 2014; Kaur et al., 2020; Lira et al., 2020). With increasing urbanization, ensuring the quality control of wastewater discharged into water bodies has become a significant challenge, particularly in developing countries where gaps in public health surveillance hinder the provision of adequate sanitation services in cities (Abraham, 2011; Ercumen et al., 2018; Iskandar et al., 2021).

In this context, the One Health approach assumes contemporary importance by recognizing the interconnectedness of human, animal, and environmental health. It seeks to promote an integrated assessment to understand and address health issues affecting humans, animals, and ecosystems. The fundamental idea is that the health of one species is intrinsically linked to the health of others and the environment they share (Mackenzie and Jeggo, 2019). In the context of AMR, One Health can relate to the presence of multidrug-resistant bacteria in soil, affecting human health through contamination of vegetables and animals traded in the food industry (Velazquez-Meza et al., 2022; Rahman et al., 2021; Kaviani et al., 2022).

Aligned with our study, the potential contamination of aquatic biota in Baía do Guajará by MDR bacteria and sanitary waste could impact human health, particularly given the extensive commercialization of fish caught in the bay for the local population.

Considering the aforementioned scenario, this study characterized the microbiome structure present in the sewage channel, highlighting the high predominance of components from the families *Aeromonadaceae*, *Enterobacteriaceae*, and *Moraxellaceae*. These families encompass genera frequently associated with foodborne and nosocomial infections, such as *Aeromonas*, *Acinetobacter*, *Enterobacter*, and *Klebsiella* (Singh et al., 2020), which were quantitatively more represented in the studied environment.

The presence of species like *Enterococcus faecium*, *Staphylococcus aureus*, *Klebsiella pneumoniae*, *Acinetobacter baumannii*, *Pseudomonas aeruginosa*, and *Enterobacter* spp. (ESKAPE pathogens) in sanitary effluents must be continuously monitored, as they pose a high risk to human health and are determinants of resistance to last-resort antibiotics such as beta-lactams (carbapenems), colistin, and other multiple drugs. The establishment of WWTPs represents an alternative to mitigate the spread of these organisms (Marutescu et al., 2023).

The microbiological diversity at the sewage channel collection points showed that the Shannon index was higher at points P2, P3, and P4 compared to P1, where there was a quantitatively lower abundance profile and significantly different diversity (Figure 2B). One possible explanation for this is that during the collection of samples from P1, conducted early in the morning (~8:00 AM), the channel had a low water level. As human activities began, there was an increase in the water level in the channel, which may be related to a change in the abundance profile and diversity at subsequent collection sites. This suggests that sewage is an environment that reflects circulating microorganisms in the local population. It is also important to highlight that the DAPC exhibited a significantly different microbial profile between each collection point, due to high inter-group variability.

The literature extensively documents comparisons between microbial communities from different environments and those originating from humans, using metagenomic approaches to establish a connection between humans and their external environment (Claesson et al., 2011; Shanks et al., 2013; David et al., 2014; Fisher et al., 2015). A study supporting this hypothesis compared the human microbiome in 71 US cities with that found in the sewage of those locations, finding a similarity of about 15% between the metagenomes. Notably, bacterial diversity in sewage was approximately three times higher than in fecal samples, indicating that sewage reflects the structure of microbial communities present in the surrounding human population (Newton et al., 2015).

The heatmap featuring six families with high pathogenic potential for humans demonstrates that genera such as *Klebsiella*, *Serratia*, *Salmonella*, *Pseudomonas*, *Escherichia*, *Enterobacter*, *Raoultella*, *Acinetobacter*, *Streptococcus*, *Sebaldella*, *Sneathia*, *Fusobacterium*, and *Streptobacillus* constituted a group with the highest abundances in this category of bacteria, maintaining consistent presence across all sampling sites (Figure 3). Most of these taxa, along with less prevalent ones like *Legionella* and *Vibrio*, form a group that is challenging to eradicate even by wastewater treatment plants (WWTPs), as several studies have shown their presence in treated effluents due to their ability to form biofilms and resist detergent action and unfavorable environmental conditions (Molofsky and Swanson, 2004; Numberger et al., 2019; Alam et al., 2021).

### 4.2 Resistome analysis

Antimicrobial resistance represents a serious contemporary issue, as patients with complex infections often exhibit low responsiveness to antibiotic therapy due to the presence of multidrug-resistant bacterial strains. This study identified a substantial number of antimicrobial resistance genes with this characteristic, with the multidrug-resistant category being the most prevalent across all sampling points.

Recent literature indicates a high prevalence of MDR microbial strains being disseminated in the environment, particularly through sewage systems, with certain species contributing significantly to the spread of these multidrug-resistant genes. These species include *Salmonella* spp., *Shigella* spp., *Staphylococcus aureus*, *Enterococcus* spp., and *Aeromonas* spp. The classes most commonly associated with multidrug resistance are glycylcyclines (GLC), beta-lactams, tetracyclines, and aminoglycosides (Adugna and Sivalingam, 2022; Zieliński et al., 2020; Gotkowska-Płachta, 2021; Harnisz and Korzeniewska, 2018).

Among the most prevalent genes, notable examples include *qacEdelta1* encoding efflux pumps resistant to acrimin and biocides (Pastrana-Carrasco et al., 2012); *mdtE* and *mdtF* encoding efflux pumps resistant to macrolides, fluoroquinolones, and penems (Zhang et al., 2011); and *bla_OXA-10_* and *bla_GES-5_*, which produce beta-lactamases that inactivate carbapenems, cephalosporins, and penems (Maurya et al., 2017; Ribeiro et al., 2014).

A wide spectrum of ARGs encoding beta-lactamases and members of the bla gene cluster was found, totaling 18 genes, including *bla_KPC_*, *bla_TEM_*, *bla_GES_*, *bla_OXA_*, *bla_CTX-M_*, and *bla_CFxA_*, which are common in Gram-negative bacteria (Ojdana et al., 2014). In this study, these genes were present in all sampling points except *bla_CTX-M_*, which was restricted to site P3. This group of genes encompasses enzymes conferring resistance to penicillins and cephalosporins, as well as extended-spectrum beta-lactamases (ESBLs), which are clinically highly relevant (Bradford, 2001; Worthington and Melander, 2014).

A wide range of bla ESBL gene variants are already known, such as *bla_CTX-M-1_*, *bla*_-GES_, and *bla_OXA-10_*, among others (Wang et al., 2015). In our data, CARD identified 155 molecular variants originating or not from different bla gene families, showing that sewage can harbor a diverse and complex resistome (Tello et al., 2012; Xiong et al., 2015; Al Salah et al., 2022; Munk et al., 2023).

When evaluating the resistome from the perspective of resistance mechanisms, studies indicate a range of evasion systems that counteract antibiotic action. In our study, it was observed that mechanisms such as enzymatic inactivation linked to beta-lactamases, efflux pumps, and molecular target modification gathered a large number of ARGs (Figure 5B). Studies show that the most prevalent mechanisms in ESKAPE group bacteria include enzymatic inactivation, modification of the molecular target due to mutations, alteration of cell permeability through regulation of porin channel expression, upregulation of efflux pumps, and mechanical protection. In addition, biofilm formation on surfaces can ensure greater survival of the organism in the environment, including protection against antibiotics (Santajit and Indrawattana, 2016; De Oliveira et al., 2020).

From the analysis of contigs from four pathogens listed in the CDC’s threat ranking, it was found that they exhibit characteristics similar to those described in the 2019 report, with varied multidrug-resistant profiles highly capable of causing severe infections in humans. Precise investigations like this are crucial for understanding the resistome structure of important bacterial genera, providing additional knowledge to the scientific community and the public to collectively combat AMR. Within this context, as a global leader in antibiotic consumption and production, China implemented a new national action plan in 2022 aimed at reducing the spread of antibiotic resistance in the country by 2025, through collaboration across various public and private sectors following the One Health model (Chen et al., 2023). Therefore, the Brazilian government must pay more attention to the country’s health conditions, invest in health education for the population, develop new guidelines for antibiotic therapy and base itself on international models for combating AMR and adapt them to the reality of Brazil so that antimicrobial resistance can be contained.

Finally, our study interestingly demonstrated that the distribution of antimicrobial classes and different categories of resistance mechanisms was highly heterogeneous, as both components were present across all sampling points (Figures 5C,D). This can be attributed to the interconnected sewage system in the city of Belém, which consolidates effluents from multiple sources in specific locations, such as the study site. Therefore, it is important to highlight that the discharge of hazardous materials into the city’s sewage system, including hospital and industrial effluents, should undergo prior treatment to prevent amplifying the issue of AMR in the aquatic environment, particularly due to the absence of WWTPs in this area.

## 5 Conclusion

This study highlights the critical issue of antimicrobial resistance in an untreated sewage channel that discharges contaminants into the bay, compromising water quality and public health. The presence of fecal coliforms and pathogenic bacteria, such as those from the *Enterobacteriaceae* family, indicates significant risks of fecal contamination and disease transmission, exacerbated by inadequate sanitation infrastructure.

Our findings reveal a diverse resistome with a high prevalence of multidrug resistance genes, posing substantial challenges to contemporary medicine due to the reduced efficacy of antibiotics against MDR bacterial strains. The study also highlights the role of sewage systems in the spread of AMR, with a notable abundance of genes encoding beta-lactamases, efflux pumps, and other resistance mechanisms. This suggests the need for improved wastewater treatment strategies, especially for hospital and industrial effluents, to reduce AMR in the aquatic environment. Despite limitations such as small sample size and lack of comparisons between different environments such as pre- and post-WWTP microbiome analysis, this exploratory study provides important insights into the dynamics of AMR and the need to use a One Health approach to address this issue in a multifaceted way.

## Data Availability

The datasets presented in this study can be found in online repositories. The names of the repository/repositories and accession number(s) can be found in the article/Supplementary material.
