# Supplementary material for: Metagenomics insights into bacterial diversity and antibiotic resistome of the sewage in the city of Belém, Pará, Brazil
Source: Front Microbiol. 2024 Nov 19;15:1466353. doi: 10.3389/fmicb.2024.1466353 (PMC11611572; doi:10.3389/fmicb.2024.1466353)
Supplement: Supplementary file 1 [file Table_1.DOCX]

Supplementary Material

| **Parameter** | **Result** | **Unit** |
| --- | --- | --- |
| Dissolved oxigen | 6.07 | mg/L |
| Salinity | 1910 | mg/L |
| pH | 1.17 | à 25°C |
| Condutivity | 407 | μS/cm à 25°C |
| Chemical-oxygen demand | 112.60 | mg/L |
| Biochemical-oxygen demand | 32.64 | mg/L |
| Total coliform | 1600 | NMP/100 mL |
| *Escherichia coli* | Presence | /100mL |

Supplementary Table 1. Physical-chemistry parameters.

| **Parameter** | **P1R1** | **P1R2** | **P1R3** | **P2R1** | **P2R2** | **P2R3** |
| --- | --- | --- | --- | --- | --- | --- |
| Sites | P1 | P1 | P1 | P2 | P2 | P2 |
| Total of reads | 23709522 | 22655023 | 36598809 | 21165980 | 58020424 | 24207568 |
| Mean of reads (per site) |  | 27654451,33 |  |  | 34464657,33 |  |
| Contigs | 341064 | 376893 | 565648 | 348868 | 526260 | 316018 |
| Min (pb) | 200 | 200 | 200 | 200 | 200 | 200 |
| Max (pb) | 30435 | 42788 | 168335 | 33689 | 144709 | 42619 |
| Mean read length (pb) | 612 | 668 | 693 | 655 | 671 | 645 |
| N50 | 629 | 728 | 743 | 702 | 712 | 689 |
| CARD mean identity (%) | 99.09 | 99.58 | 99.52 | 99.48 | 99.35 | 99.5 |
| CARD mean length (%) | 99.67 | 99.53 | 99.02 | 99.54 | 98.87 | 99.67 |
| **Parameter** | **P3R1** | **P3R2** | **P3R3** | **P4R1** | **P4R2** | **P4R3** |
| Sites | P3 | P3 | P3 | P4 | P4 | P4 |
| Total of reads | 29571539 | 75197478 | 24564557 | 72504473 | 20413311 | 24347148 |
| Mean of reads (per site) |  | 43111191,33 |  |  | 39088310,67 |  |
| Contigs | 335187 | 727428 | 295576 | 694782 | 270613 | 276519 |
| Min (pb) | 200 | 200 | 200 | 200 | 200 | 200 |
| Max (pb) | 37093 | 94996 | 32248 | 251389 | 88099 | 64079 |
| Mean read length (pb) | 637 | 664 | 654 | 692 | 673 | 685 |
| N50 | 692 | 706 | 703 | 750 | 735 | 752 |
| CARD mean identity (%) | 99.53 | 99.34 | 99.3 | 98.88 | 99.43 | 98.93 |
| CARD mean length (%) | 99.25 | 98.56 | 98.62 | 98.47 | 99.1 | 97.42 |

Supplementary Table 2. Reads-sequenced statistics from metagenomic data obtained from each sampling site (P1, P2, P3, and P4).

| **Site** | **Sample** | **Richness** | **Equability (Pielou)** | **Shannon** |
| --- | --- | --- | --- | --- |
| P1 | P1R1 | 7250 | 0.5183 | 4.6073 |
| P1 | P1R2 | 7195 | 0.4713 | 4.1855 |
| P1 | P1R3 | 7370 | 0.4736 | 4.2173 |
| P2 | P2R1 | 7258 | 0.5221 | 4.6415 |
| P2 | P2R2 | 7467 | 0.5243 | 4.6761 |
| P2 | P2R3 | 7253 | 0.5423 | 4.8202 |
| P3 | P3R1 | 7226 | 0.5276 | 4.6878 |
| P3 | P3R2 | 7512 | 0.534 | 4.7652 |
| P3 | P3R3 | 7267 | 0.5412 | 4.812 |
| P4 | P4R1 | 7517 | 0.5342 | 4.7675 |
| P4 | P4R2 | 7171 | 0.5558 | 4.9344 |
| P4 | P4R3 | 7225 | 0.5408 | 4.8054 |

Supplementary Table 3. Ecological parameters of reads-sequenced from each sampling site (P1, P2, P3, and P4).
